# Frozen mountain pine needles: The endodermis discriminates between the ice‐containing central tissue and the ice‐free fully functional mesophyll

**DOI:** 10.1111/ppl.13865

**Published:** 2023-02-08

**Authors:** Matthias Stegner, Othmar Buchner, Michael Geßlbauer, Jasmin Lindner, Alexander Flörl, Nannan Xiao, Andreas Holzinger, Notburga Gierlinger, Gilbert Neuner

**Affiliations:** ^1^ Department of Botany University of Innsbruck Innsbruck Austria; ^2^ Institute of Biophysics, University of Natural Resources and Life Sciences (BOKU) Vienna Austria

## Abstract

Conifer (Pinaceae) needles are the most frost‐hardy leaves. During needle freezing, the exceptional leaf anatomy, where an endodermis separates the mesophyll from the vascular tissue, could have consequences for ice management and photosynthesis. The eco‐physiological importance of needle freezing behaviour was evaluated based on the measured natural freezing strain at the alpine treeline. Ice localisation and cellular responses to ice were investigated in mountain pine needles by cryo‐microscopic techniques. Their consequences for photosynthetic activity were assessed by gas exchange measurements. The freezing response was related to the microchemistry of cell walls investigated by Raman microscopy. In frozen needles, ice was confined to the central vascular cylinder bordered by the endodermis. The endodermal cell walls were lignified. In the ice‐free mesophyll, cells showed no freeze‐dehydration and were found photosynthetically active. Mesophyll cells had lignified tangential cell walls, which adds rigidity. Ice barriers in mountain pine needles seem to be realised by a specific lignification patterning of cell walls. This, additionally, impedes freeze‐dehydration of mesophyll cells and enables gas exchange of frozen needles. At the treeline, where freezing is a dominant environmental factor, the elaborate needle freezing pattern appears of ecological importance.

## 1 INTRODUCTION

The ability to adapt and to survive freezing temperatures is a key factor in plant distribution to specific geographical areas. The frost‐hardiest conifer species are in the northern hemisphere, where the coldest climatic zones with long‐lasting winter periods are found (Bannister & Neuner, 2001). There, Pinaceae are most successful at arctic and high‐elevation climatic treelines (Becwar et al., 1981; Körner, 2012) and commonly dominate boreal forests (Givnish, 2002). Species from this family have evolved evergreen needle‐like leaves that are among the most frost‐hardy leaves. Mountain pine needles survive temperatures down to −85.0 ± 8.1°C (Bannister & Neuner, 2001). This exceeds by far the lowest air temperatures recorded at the treeline in the Alps and further above in the alpine life zone (Neuner, 2014). However, during winter, the frequency and duration of frosts may be even more incriminatory than their absolute severity. While conifer stem (Charrier et al., 2015; Mayr et al., 2006) and bud (Kuprian et al., 2017) temperatures recorded at the alpine timberline indicate prolonged frost periods and frequent freeze/thaw cycles, little is known about how needles freeze in nature. Although pine needles survive these extraordinary temperature conditions, the underlying freezing resistance mechanism remains unclear.

Mountain pine needles have an elaborate, highly xeromorphic anatomy (Esau, 1977) typical for all Pinaceae (*Abies*, *Larix*, *Picea*, and *Pinus*). The mesophyll is separated from the central vascular cylinder by a distinctly differentiated, thick‐walled endodermis. Within the endodermis, there are one or two vascular bundles embedded in the transfusion tissue. Transfusion tracheids transport water radially. Strasburger cells transport assimilates from the mesophyll to the phloem. Transfusion parenchyma cells scavenge solutes from transpiration water (Canny, 1993). Mesophyll cells are called arm palisade parenchyma cells because their cell walls have internal ridges projecting into the cell lumina. Leaf anatomy significantly affects freezing behaviour (Ball et al., 2004; Bertel et al., 2021; Hacker et al., 2008; Hacker & Neuner, 2008; McCully et al., 2004). The specific function of these conifer‐specific anatomical structures, and their role in freezing behaviour and management of extracellular ice, and their effect on overall frost tolerance are largely unknown.

Some attempts have been made to investigate the freezing behaviour of conifer needles. In *Picea abies*, the ice enters the needle via the xylem from the stem (Kuprian et al., 2018). An ice nucleation via the central vascular cylinder was already supposed in early studies about freezing behaviour of conifer needles (Kaku, 1971; Kaku & Salt, 1968; Salt & Kaku, 1967). Based on a particular form of freezing exotherms, it has been suggested that the radial growth of ice out of the frozen vascular tissue is slowed by the endodermis (Kaku, 1971; Kaku & Salt, 1968; Salt & Kaku, 1967). The high‐speed freezing rates (−1°C min^−1^) in these experiments may, however, not allow drawing conclusions about freezing under natural conditions. In a cryo‐SEM study, freezing of cold‐acclimated *Pinus radiata* did not change the shape of the needles or the relative areas occupied by the central vascular cylinder and mesophyll and the endodermis was no ice barrier (Roden et al., 2009). Cell types responded very differently when frozen down to −11°C. Mesophyll cells shrank, exhibiting freeze‐dehydration. Extracellular ice accumulated in the mesophyll but gas spaces remained during freezing. In the central vascular cylinder, ice accumulated in transfusion tracheids, which expanded to occupy areas made vacant by shrinkage of transfusion parenchyma, Strasburger cells and the endodermis. However, how ice is managed during long frozen periods in much frost‐hardier conifers needles is unclear. In *P. abies*, buds ice barriers are realised by specific substances reducing the cell wall pore diameter (Kuprian et al., 2017) as the freezing point of water in narrow cell wall pores is depressed. In herbaceous leaves of *Ranunculus glacialis*, especially cells in close vicinity to apoplastic ice masses revealed accumulations of triglycerides (Stegner et al., 2020b). We expect that in extremely frost‐hardy conifer needles, the different cells and tissues correlate with characteristics that might be optimised for different functionalities playing a role in freezing behaviour.

As ice masses in intercellular spaces inhibit gas diffusion, prolonged periods of cold and freezing may affect or even block needle gas exchange. Photosynthesis of evergreens in winter is mostly inactivated (Verhoeven, 2013). Nevertheless, during warm periods in winter, needles exhibit low rates of photosynthesis (Sevanto et al., 2006) and yet, the respiration necessary for basic physiological processes may not completely stop. Observations on gas exchange of conifer needles during freezing indicate a biochemical limitation of photosynthesis rather than a limitation by stomatal closure (Gaumont‐Guay et al., 2003; Strand et al., 2002; Strand & Öquist, 1985). Large gas spaces in the mesophyll of frozen needles of *P. radiata* (Roden et al., 2009) might indicate that gas exchange in frozen conifer needles may not be completely blocked. Whether ice‐free intercellular spaces exist in the mesophyll of needles of the frost‐hardiest conifers and allow gas exchange in the frozen state is unknown.

For mountain pine needles, we aimed to assess the specific freezing behaviour responses of the highly differentiated cells and tissues to ice formation and the consequences of ice formation on gas exchange. We addressed the following questions: How frequent, for how long and at what freezing temperature does ice form in this extremely frost‐hardy conifer needles at the alpine treeline? How does ice propagate in needles and where is it localised? Which tissues in the needle freeze‐dehydrate, shrink or retain their shape upon freezing? Can some molecular cell wall components have an effect on cell wall properties, which in turn affect the needle freezing behaviour? What are the consequences of freezing behaviour and ice formation for needle respiratory and photosynthetic gas exchange?

## 2 MATERIALS AND METHODS

### 2.1 Plant material and study site

Mountain pine (*Pinus mugo* Turra) naturally grows on Mt. Patscherkofel within the area of the Alpine Garden of the University of Innsbruck (1919 m a.s.l., 47°12′39.20″N, 11°27′1.97″ E). The Alpine Garden is situated within the treeline ecotone in the alpine zone. The experiments were conducted during fall, winter and spring; consequently, the plants were in a cold hardened state. Twigs (approx. 20 cm) with attached needles were cut, stored in a cooler bag and transported to the respective laboratories. For gas exchange measurements, mountain pine branches were collected from potted plants.

### 2.2 Needle freezing at the alpine treeline

At the investigation site, micrometeorological data was recorded from 5 October 2018 till 27 June 2019 with a climate station (CR1000, Campbell Scientific). The soil temperature was recorded in a depth of 5 cm. Photosynthetic active photon flux density was measured with a quantum sensor (SKP215, Campbell Scientific). Needle temperature records were done with copper constantan thermocouples (TT‐TI‐36, Omega Engineering Inc.). To ensure good contact, the thermocouple tips were glued to the needles with cyanoacrylate glue (Super Glue Control, Uhu). The harsh weather conditions required high maintenance and resulted in an unequal and changing number of temperature replications (*n* ≥ 5). Additionally, air temperatures in close vicinity to the leaves were measured as references. While a measurement frequency of one measurement every 5 min was used in the above +5°C range, data were monitored at a high frequency (0.5 s^−1^) at lower temperatures to detect needle‐freezing events. The climate station data was uploaded automatically every 6 h to a server, which allowed online data monitoring.

**FIGURE 1:**
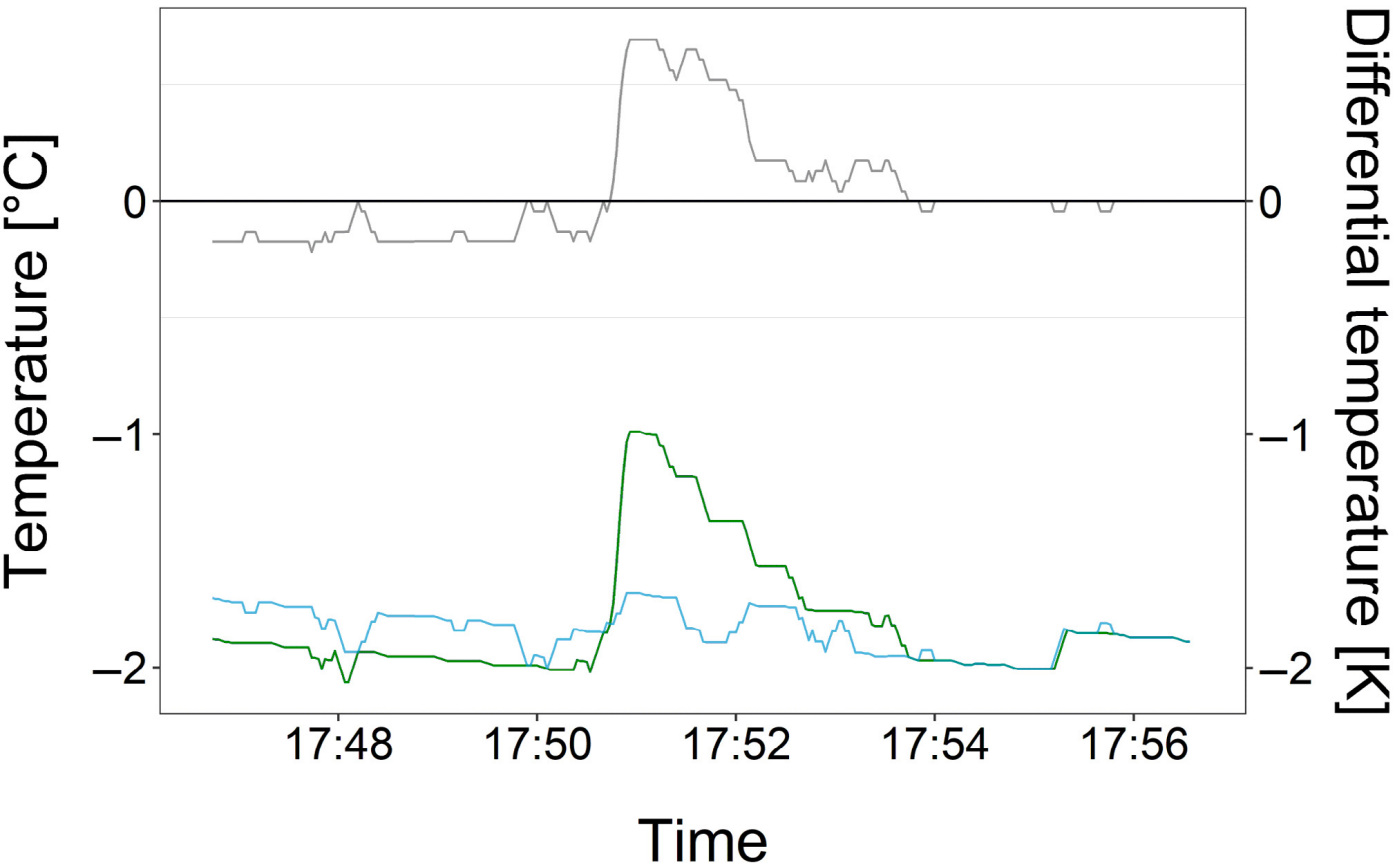
Individual leaf freezing was detected by high‐frequency temperature measurements of mountain pine leaves (green line) compared to a reference temperature sensor (blue line) in close vicinity to the leaves. The reference temperature was subtracted from the leaf temperature and yielded the differential temperature (grey line), which was used for computer‐aided detection of freezing exotherms. Rolling means of 4 temperature records were calculated to minimise noise due to minor temperature fluctuations.

During phase transition from liquid water to ice, crystallisation heat is released; so‐called freezing exotherms. Freezing exotherms can be detected by subtracting the temperature in close vicinity to the leaf from the actual leaf temperature; which is related to differential thermal analysis (DTA) (Burke et al., 1976). With the high‐frequency temperature data, single‐leaf freezing exotherms could be as well detected in the field. Freezing exotherms were detected computer‐aided and finally checked and verified by eye. During ice nucleation, the needle temperature (green line; Figure 1) showed a rapid increase until a maximum and then gradually decreased towards the reference ambient temperature (Figure 1). By subtraction of the reference air temperature (blue line; Figure 1) from the needle temperature, a differential temperature (DTA temperature; grey line; Figure 1) was calculated, which allowed identification of leaf freezing events. The ice nucleation temperatures presented are the temperatures immediately before the onset of freezing.

### 2.3 Raman imaging and histochemical staining

Raman spectroscopy was applied to probe the chemical composition within cross‐ and longitudinal‐sections of mountain pine needles. By this, the chemistry can be pictured in context with the microstructure (Gierlinger, 2018) and the results were compared to histochemical staining methods. Sections were cut with a thickness of 20 μm for cross‐sections and 35 μm for longitudinal sections and used for Raman imaging and histochemical staining. Fuchsin–Chrysoidine–Astrablue staining was performed according to Henkel (2003) with 5 min immersion in Fuchsin–Chrysoidine–Astrablue solution and Phloroglucinol staining as described in Yeung (1998). Stained sections were embedded in Euparal and photographed with a Labophot‐2 microscope (Nikon Corporation). The subsequent sections used for Raman imaging were investigated native and after washing in EtOH for 10 min to reduce sample fluorescence. For Raman imaging, the sections were placed on glass slides with a droplet of D_2_O, closed by a cover slip and sealed with nail polish. We used a confocal Raman microscope (Alpha300RA, WITec GmbH) equipped with a 532 nm laser line, a spectrometer (600 g mm^−1^ grating, UHTS 300, WITec, GmbH), a CCD camera (DV401A‐BV‐351, Andor) and a 100x oil immersion objective (numerical aperture = 1.4, coverslip corrected 0.17 mm; Carl Zeiss AG). Microsections were scanned with a laser power of 20–30 mW and one full spectrum (100–3780 cm^−1^) was acquired at every pixel with a step size of 333 nm and an integration time of 0.1–0.4 s. Microsections from three different needles were scanned, whereby long overview runs were included as well as zooms into selected regions. The best representative overview scans from one cross‐section and one EtOH‐washed longitudinal‐section are exemplarily shown. From all hyperspectral datasets, cosmic rays were removed and the baseline corrected before generating chemical images based on True Component analysis using WITec project PLUS 5.2 software. This software locates the most different spectra within the mapped area and calculates every pixel as a linear combination of the established component spectra with a basis analysis algorithm (Dieing & Ibach, 2010). The results are false colour images of the different components and the corresponding component spectra. We increased the number of components as long as meaningful different component spectra were derived. For verification of lignin composition and amount, average spectra were extracted from selected needle cell walls (xylem, endodermis and parenchyma) and compared to the average spectra acquired from cell walls of pine stem wood.

### 2.4 Needle freezing in laboratory experiments

Laboratory freezing treatments were conducted inside of cooling compartments of commercial chest freezers (Neuner et al., 2020). Temperature runs are freely programmable. Cooling rates were close to natural and at a maximum rate of −3 K h^−1^. Ice nucleation was triggered at natural freezing temperatures either by ice nucleation active bacteria (*Pseudomonas syringae* van Hall 1902) or by external ice crystal application (Buchner et al., 2020). The sample temperature was monitored with thermocouples (TT‐TI‐36, Omega Engineering Inc.).

### 2.5 Infrared differential thermal analysis

Freezing of mountain pine branches (*n* = 3) and attached leaves was monitored with a digital infrared camera (ThermaCAM S60, FLIR Systems). Subtracting the infrared image prior to the freezing event from the following images enabled to spatiotemporally visualise freezing. This procedure is called infrared differential thermal analysis (IDTA) (Hacker & Neuner, 2007) and is related to DTA (Burke et al., 1976). For details about the experimental setup please see Hacker and Neuner (2007) and Stegner et al. (2019). To elucidate how the central vascular cylinder or the mesophyll freezes and in what temporal order, we used an experimental variation: the mesophyll of the needles was partly removed by a longitudinal cut with a razor blade to directly monitor freezing processes in the central vascular cylinder (*n* = 12) with the infrared camera.

### 2.6 Cryo‐microscopy in reflected‐polarised‐light

To visualise in which tissues ice crystals are accommodated during sub‐lethal freezing, cryo‐microscopy in reflected‐polarised‐light was applied (Stegner, Wagner, & Neuner, 2020a). Cryo‐microscopy in reflected‐polarised‐light distinguishes ice from liquid water by the use of polarised light. The system is placed in a fully temperature‐controllable environment. Twigs with needles attached (*n* = 8) were placed in this environment and gradually exposed to freezing temperatures. Sectioning was done either transversally or longitudinally with a microtome (GSL1, Schenkung Dapples) and the cut surface was immediately inspected.

### 2.7 Semi‐thin sectioning of high‐pressure frozen samples

Needles from individual twigs (*n* = 3) were continuously cooled to sub‐lethal temperatures and fixed before ice nucleation at −2°C and in the frozen state at −6°C/−15°C. For fixation, the samples (*n* = 3) were transferred to an automatic freezing unit according to a previously published protocol, which prevents samples from thawing during the transfer (Buchner et al., 2020). High‐pressure freeze fixation was performed by a Leica Empact high‐pressure freezer; after that, substitution took place in a Leica EM AFS. Freeze substitution was performed in −80°C acetone containing 2% OsO_4_ and 0.05% uranyl acetate (Buchner et al., 2020). The samples were epoxy resin embedded and heat‐polymerised. Semi‐thin sections (0.6 μm) were prepared at a Reichert Ultracut S (Leica Microsystems). The sections were transferred onto glass slides and stained with 0.3% Toluidine Blue O (Holzinger et al., 2009), investigated with an Axiovert 200 M microscope and images were captured with an AxioCam HRc (Carl Zeiss).

### 2.8 Gas exchange of frozen needles

Gas exchange of mountain pine needles was measured before, during, and after ice formation with a GFS3000 gas exchange measurement system (Walz). Three terminal shoots were detached and put into vials filled with tap water and placed inside a temperature‐controlled freezer. There, 6–8 needles were sealed into the standard gas exchange measurement cuvette (3010‐A, Walz) that, as a whole, was inside the freezer. It was possible to cool the needles inside the cuvette until the freezing took place and beyond. Needle temperature and the net photosynthesis rate were measured simultaneously throughout cooling. To allow photosynthesis and to keep the stomata open, the needles were irradiated with a moderate photosynthetic active photon flux density of 100 μmol photons m^−2^ s^−1^. Before and after cooling, the maximum quantum efficiency of photosystem 2 (PS II) photochemistry (Fv/Fm) was determined (LED array PAM fluorometer 3055‐FL, Walz). To prevent measuring errors due to the constant temperature change, the internal infrared gas analyser of the GFS 3000 system was calibrated in 6 min intervals. Needle temperature monitoring allowed the detection of needle freezing by exotherm detection. Furthermore, the set parameters were: Flow 750 μmol s^−1^, CO_2_ 400 ppm, H_2_O 4000 ppm, O 4000 ppm.

### 2.9 Data analysis

Numerical data processing and analysis were performed with R (R Development Core Team, 2020). Results are given as mean ± standard deviation.

## 3 RESULTS

### 3.1 Needle freezing in the field

**FIGURE 2:**
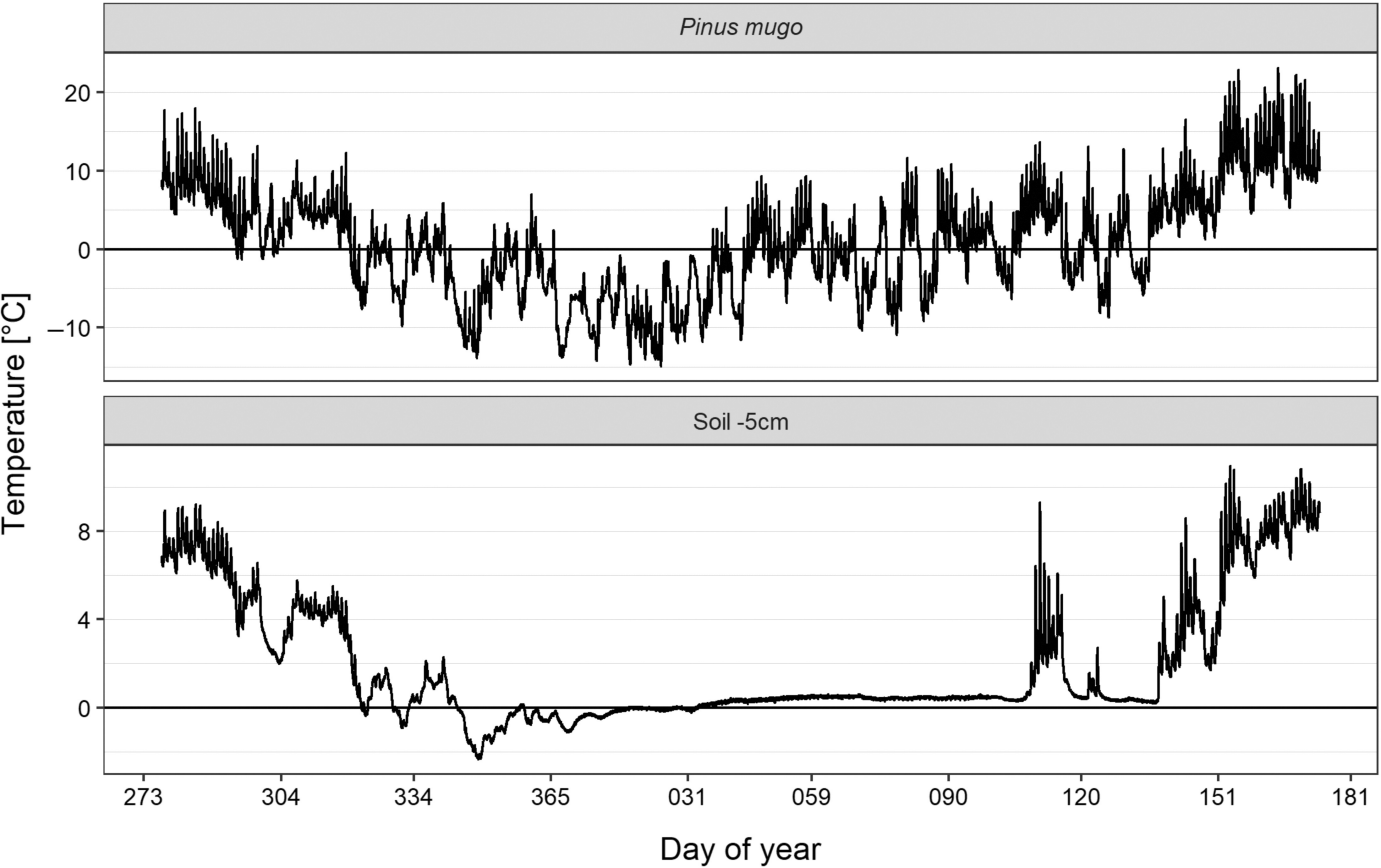
Microclimatic records revealed the actual leaf freezing strain at the subalpine investigation site (1919 m a.s.l.). Leaf temperatures of mountain pine remained lower than 0°C for long time periods. The soil temperature was only moderately low (minimum temperature −2.3°C) compared to the needle temperatures. Limitation of water uptake due to frozen soil occurred only for a short period of time.

Needle freezing temperatures were detected for about seven months between 21 October 2018 (day of year 294) and 16 May 2019 (day of year 136) (Figure 2). Mountain pine needles experienced 72 ice days (Figure 2). Individual needles of mountain pine stayed permanently frozen for a maximum of 37 days. Below snow, the soil was moderately frozen (minimum temperature − 2.3°C; Figure 2) and already thawed in February and remained unfrozen thereon. Consequently, periods of limited water availability were present but short during the investigation period. During the night, the needle temperatures hardly deviated from the air temperatures. Nocturnal needle temperatures of mountain pine could be at maximum −1.9°C colder than the air, but the mean temperature deviation (needle – air) was +0.0 ± 0.2°C. Consequently, radiative freezing did not play a major role for mountain pine needles at the subalpine investigation site. Ice nucleation was detected in mountain pine needles at −1.9 ± 0.5°C (*n* = 40). All detected freezing exotherms were single‐peak shaped.

### 3.2 Microchemistry of mountain pine needles

**FIGURE 3:**
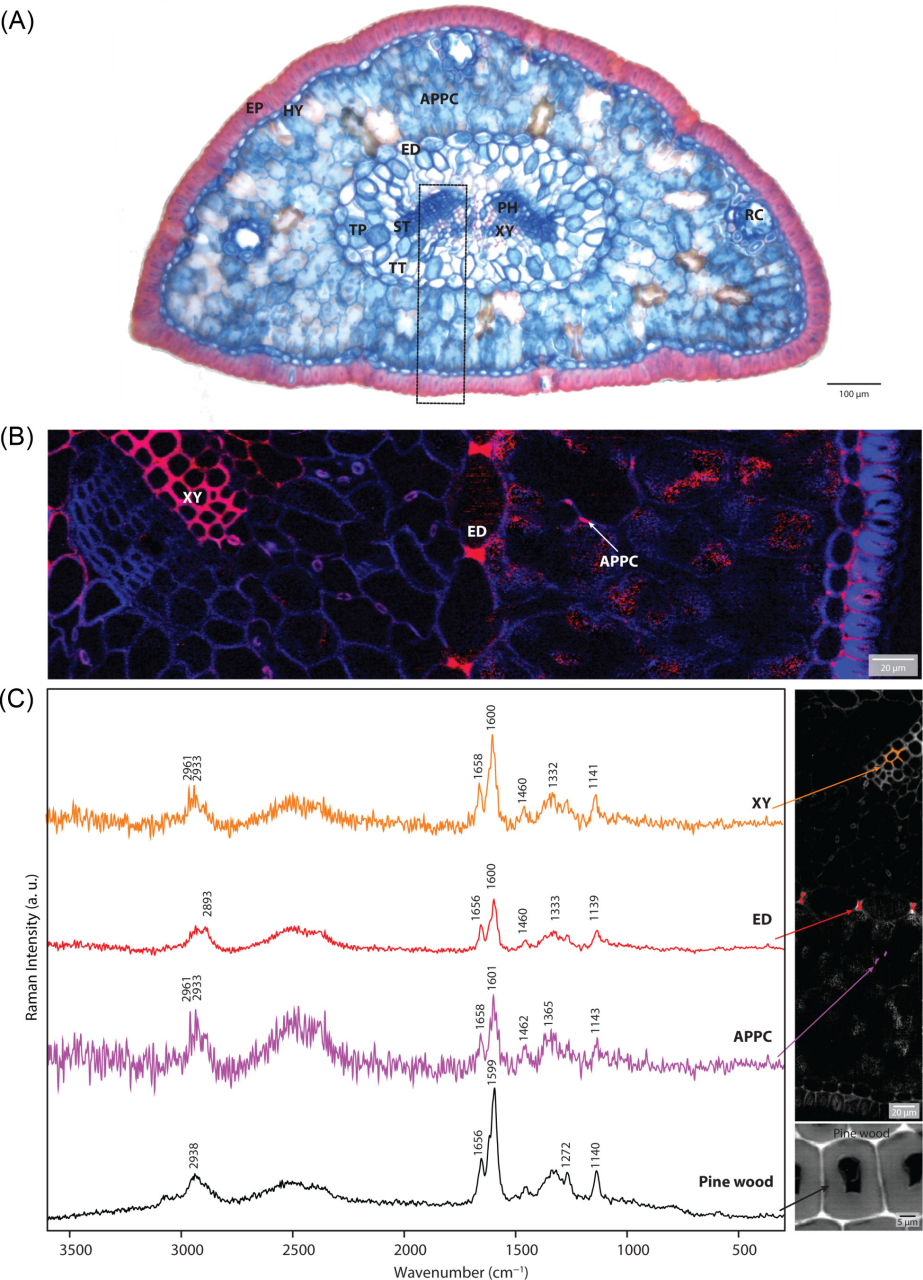
Light and confocal Raman microscopy images of mountain pine needle cross‐sections. (A) Fuchsin–Chrysoidine–Astrablue staining revealed a lignified epidermal layer (EP), the xylem (XY) in the vascular bundles, and the endodermis (ED). (B) Raman imaging (frame inserted in A) based on True Component Analysis of the cell walls (in blue) and lignified xylem, radial walls of the endodermis and partial walls of the arm palisade parenchyma cells (APPC, red), overlay of both images. (C) Extracting average spectra selectively from the xylem cells (orange), radial endodermis walls (red), the walls of the arm palisade parenchyma cells (purple) in the needle and from pine wood (black), confirmed similar lignin structures within the needle cell walls and pine wood. APPC, arm palisade parenchyma cell; ED, endodermis; EP, epidermis; HY, hypodermis; PH, phloem; RC, resin channel; ST, Strasburger cells; TP, transfusion parenchyma; TT, transfusion tracheids; XY, xylem.

The needle anatomy of mountain pine follows the characteristic anatomical pattern of conifer needles (Huber, 1947; Napp‐Zinn, 1966), which is characterised by an endodermal cell layer, the central vascular tissue, the photosynthetic active arm palisade parenchyma cells and resin channels (Figures 3A and S1). The arm palisade parenchyma cells are protected by the hypodermis and the strong epidermal layer. The two vascular bundles consist of the xylem and phloem, and are embedded within the transfusion tissue, which consists of transfusion tracheids, the transfusion parenchyma and Strasburger cells. Fuchsin–Chrysoidine–Astrablue and phloroglucinol stainings provide an overview of lignified tissues and cells (Figures 3A and S1). In both staining protocols, the outer epidermis had the most reddish‐stained cell walls and was therefore considered to have the most lignified cell walls, followed by the xylem in the vascular bundles and the endodermis. Especially with the phloroglucinol staining, the radial walls of the endodermis were stained dark red, which points to cinnamaldehyde groups of lignin (Figure S1). This exceptional lignification of the radial endodermis wall was verified by Raman imaging (Figure 3B, red). The large advantage of Raman imaging is that we can, first of all, calculate images based on spectral (= chemical) differences (Figures 3B and S2) and then extract Raman spectra from every region of interest to verify the molecular structure of each cell wall or tissue. Extracting average spectra selectively from the red‐coloured xylem cells, radial endodermis walls, the stained walls of the arm palisade parenchyma cells in the needle and pine wood confirmed similar lignin structures within the needle cell walls and pine wood (Figure 3C). The most prominent lignin bands at 1656, 1660, 1460, 1333, 1272, and 1140 cm^−1^ were found in similar ratios in all extracted average spectra and are typical bands from coniferyl alcohol and coniferyl aldehyde entities (Bock et al., 2020). Spectra of the arm palisade parenchyma cells were very noisy as high fluorescence background overlaid the Raman signal in these tissues (also note the noisy false pixels in the arm palisade parenchyma cells Figure 3B), and lignin bands were only found at a few places (Figure 3B arrows and Figure 3C).

**FIGURE 4:**
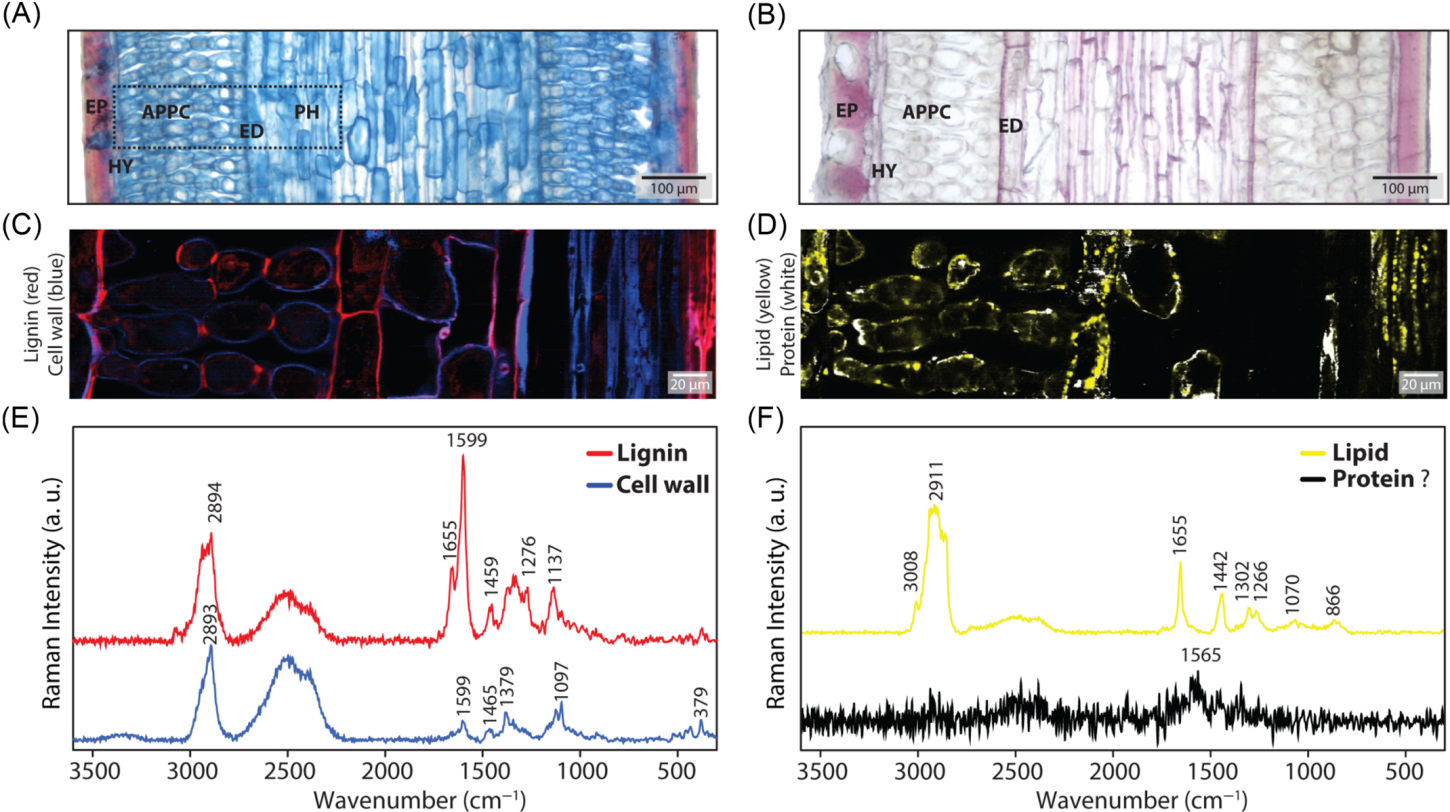
Light and confocal Raman microscopy images of longitudinal sections of mountain pine needles. (A and B) Fuchsin–Chrysoidine–Astrablue and phloroglucinol staining showed that the outer epidermal layer (EP), hypodermis (HY) and the endodermis (ED) were lignified. (C) Raman imaging (frame inserted in A) based on True Component Analysis of the cell walls (in blue) and lignified hypodermis, arm palisade parenchyma cell (APPC) walls, endodermis, and inner xylem (XY, in red), overlay of both images. By this, it was possible to resolve the special lignification pattern of the APPC, as only the connected tangential walls were lignified. (D) The lipids were visualised in the ED and in the inner phloem (PH) tissue (in yellow), while the unknown component was mainly in the cell walls of the parenchyma cells (in white). (E–F) Extracting average spectra from the regions (based on C, blue and red area; D, yellow and white area, respectively). APPC, arm palisade parenchyma cells; ED, endodermis; EP, epidermis; HY, hypodermis.

For a more comprehensive view, longitudinal sections were investigated with histochemical staining (Figure 4A,B) and Raman imaging (Figure 4C–F). Both histochemical stainings confirmed the epidermis and hypodermis to be lignified as well as the endodermis (Figure 4A,B). To get rid of the high fluorescence background and get a better signal‐to‐noise ratio of the spectra, the micro sections were also scanned after EtOH extraction (Figure 4C–F). High‐quality spectra could be derived and revealed new insights into the lignification of arm palisade parenchyma cells (Figure 4C–E). While only small wall fragments have been visualised as lignified in the cross‐sections (Figure 3C, arrows), the longitudinal section displayed a regular pattern of lignified tangential arm palisade parenchyma cell walls (Figure 4C). Only the tangential walls are lignified; consequently, the arm palisade parenchyma cells are in a strip‐like arrangement between the endodermis and the hypodermis (Figure 4A–C). The endodermis, hypodermis and the inner xylem were most lignified (red colour, Figure 4C; red spectrum Figure 4E), while the other cell wall spectra were dominated by carbohydrate bands, for example 1379, 1097 and 379 cm^−1^ (Figure 4E, blue spectrum). In the thicker longitudinal sections, a more comprehensive imaging of the cell contents was possible (Figure 4D). Lipids accumulated mainly in the endodermis and in the inner phloem tissue (Figure 4D, yellow), while another component accumulated mainly in the cell walls of the parenchyma cells (Figure 4D, white). The yellow component spectra can clearly be assigned to lipids (Figure 4F, yellow spectrum), but the white regions comprise noisy spectra with a high background signal (even after EtOH extraction) and are therefore impossible to assign; probably proteins in membranes and/or aromatic components induce the high fluorescence (Figure 4F, black spectrum).

### 3.3 Freezing behaviour of needles in the lab

**FIGURE 5:**
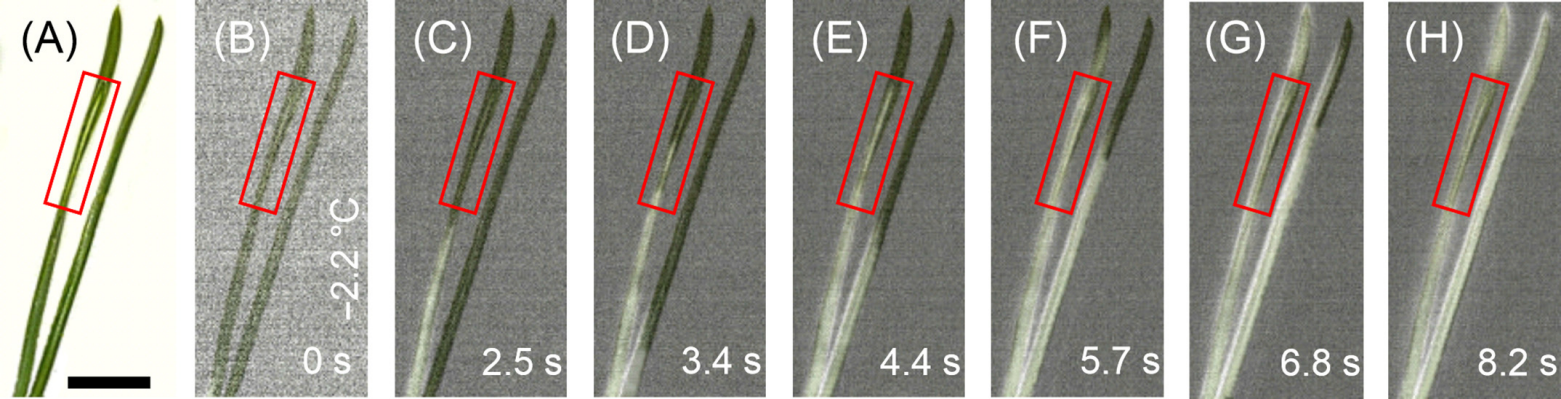
Digital image and infrared differential thermal analysis images of needles of mountain pine during controlled freezing. (A) Digital image of a needle pair of mountain pine. The right needle is intact – the left needle has been partly (red box) longitudinally sectioned to give free sight to the vascular tissue. (B–H) Image sequence of an overlay of the digital images and infrared differential thermal analysis images during controlled freezing. Whitening indicates freezing events, whereas unfrozen areas remain unchanged. Ice nucleation happened at −2.2°C in the shoot (not shown) and propagated from there into the needles. In the sectioned part of the left needle, the ice front narrows as ice spread occurs in the vascular tissue. The time span (in seconds) after initial ice nucleation is indicated at the bottom right corner. Black bar equals 1 cm.

Once the needle xylem made contact with a frozen stem sector, ice propagated into the needles (Figure S3). Ice propagated through the twigs and leaves without any obvious ice barriers (Figure S3). For the studied needles, only one freezing process could be detected by IDTA. The freezing exotherms could not be linked to a specific leaf tissue, as the method can only record surface temperatures; IDTA images thus show a heating of the whole needle. But, we could trace exothermic warming to freezing processes in the centre tissues by partly sectioning needles (Figure 5). Ice propagates longitudinally via the xylem as the ice wave passes the sectioned part of the needle (Figure 5D,E). It must be noted that a definite localisation of ice propagation is only possible at the ice front, as heat may later even capture unfrozen areas upon thermal diffusivity.

### 3.4 Localisation of ice in needles

**FIGURE 6:**
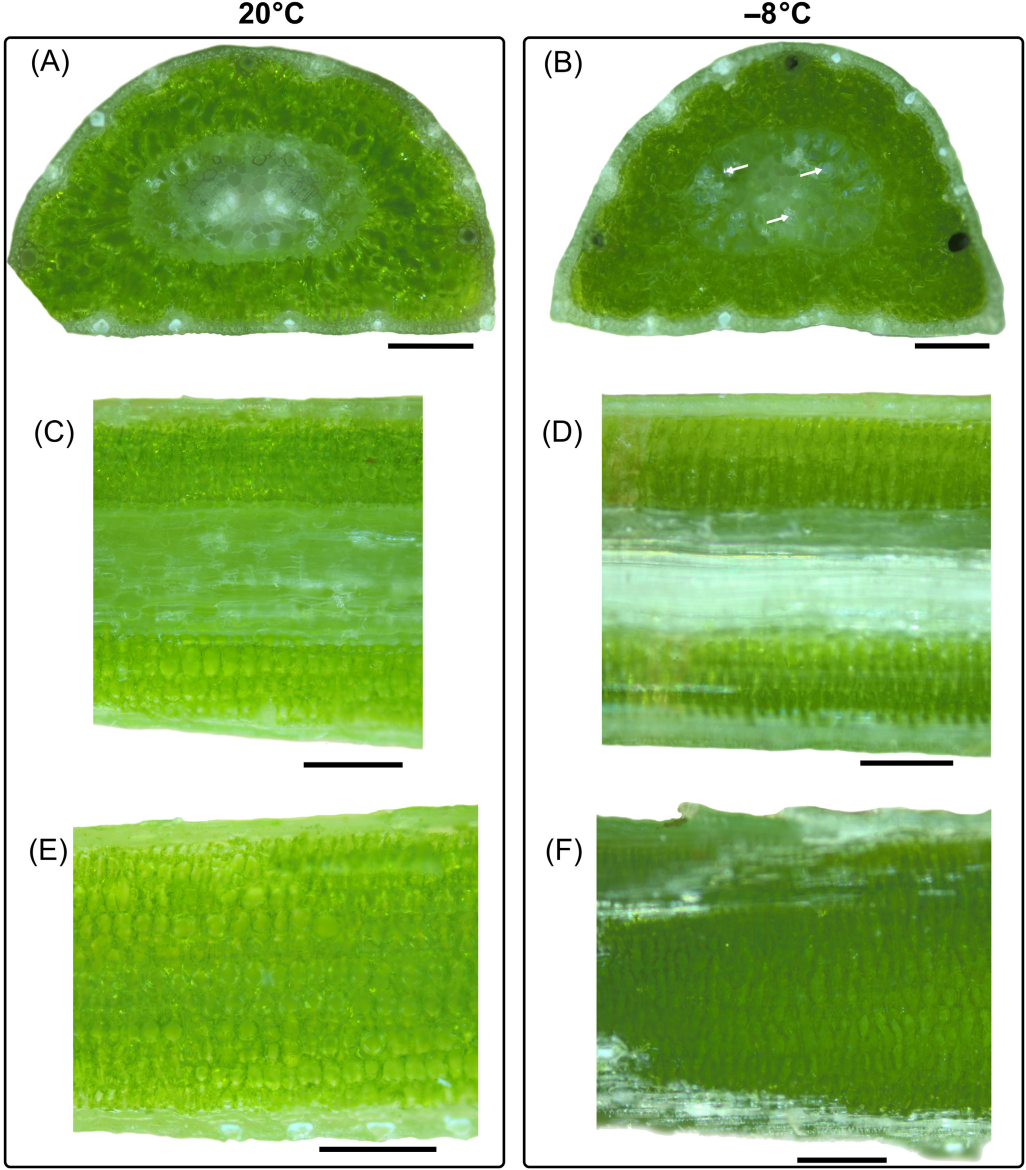
Ice localisation in needles of mountain pine. Whole needles were cross‐sectioned at 20°C (A) as controls and at −8°C (B) to localise ice by cryo‐microscopy in reflected‐polarised‐light. Ice was only detected in the vascular tissue (exemplarily indicated by white arrows). In the mesophyll tissue, no ice crystals were found. Needles were sectioned longitudinally, either through the vascular tissue (C and D) or through the mesophyll by just removing the epidermal and hypodermal tissue (E and F). Similar to the observation made in the cross‐sections, we did not detect ice crystals in the mesophyll tissue of frozen needles (D and F). Black bars equal 200 μm.

To detect ice in frozen mountain pine needles and freeze‐induced cellular changes, images of sectioned needles were compared either unfrozen (20°C) or frozen (−8°C) (Figure 6). Ice crystals were exclusively detected in the central vascular cylinder (white arrows), which best could be seen in needle cross‐sections (Figure 6A,B). The endodermis, which surrounds the transfusion and vascular tissue, shrank massively and showed freeze‐dehydration in the presence of ice. In the mesophyll tissue of cross‐sectioned mountain pine needles, we did not expect, nor could we find, ice crystals due to lacking intercellular spaces. The same was seen in longitudinal needle‐sections (Figure 6D,F), where intercellular spaces between the arm palisade parenchyma cells were free of ice crystals. In contrast, the tissues within the endodermis changed to a milky white colour when frozen, which is due to ice‐containing transfusion and xylem tracheids (Figure 6C,D). In longitudinal sections close to the needle surface, solely arm palisade parenchyma cells tissue can be seen (Figure 6E,F), again without ice in the mesophyll.

### 3.5 Cellular changes upon ice formation

**FIGURE 7:**
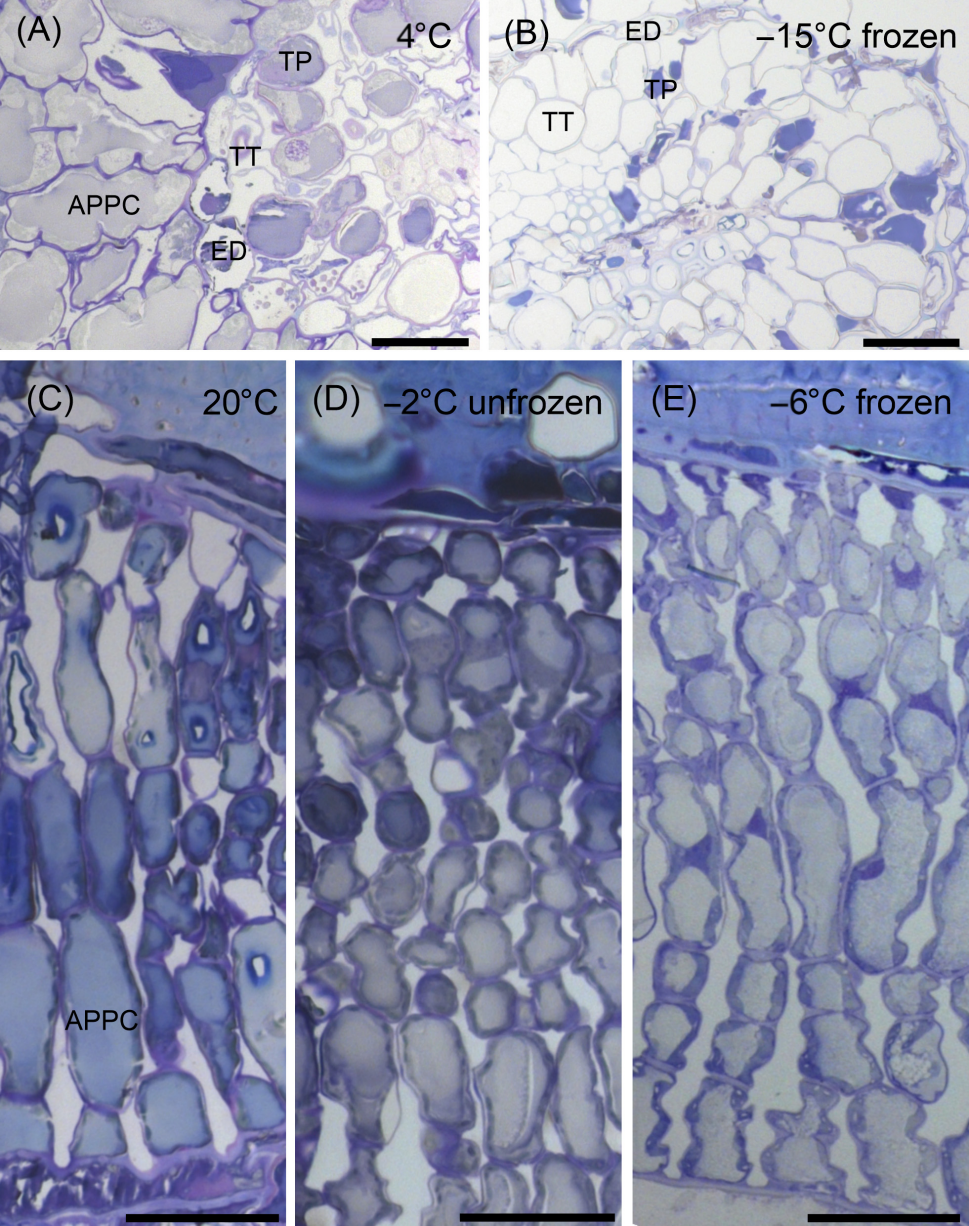
Semi‐thin sections of mountain pine needles, which were fixed at non‐freezing and (sub‐lethal) freezing temperatures. Comparison of cross‐sections of unfrozen (A) and frozen needles (B) revealed significant cellular changes upon the formation of ice. As there is no intercellular space, ice formed in the xylem and transfusion tracheids (TT). Most significant ice formation in the TT caused a distinct swelling of TT. The endodermis cells (ED) and the transfusion parenchyma (TP) shrank, that is, got freeze‐dehydrated. We could not detect any changes in the cell shape of arm palisade parenchyma cells (APPCs, longitudinal sections in C–E) when frozen. APPC, arm palisade parenchyma cell; ED, endodermis; TP, transfusion parenchyma; TT, transfusion tracheids. Black bars equal 50 μm.

Semi‐thin sections of frozen‐fixed mountain pine needles were investigated for cellular changes induced by freezing. Most significant changes were observed in the endodermis and the transfusion tissues when unfrozen controls (Figure 7A) and −15°C frozen samples (Figure 7B) were compared. The endodermis cells of frozen samples were severely freeze‐dehydrated when compared to their unfrozen turgescent appearance. This corroborates the observations by cryo‐microscopy in reflected‐polarised‐light (Figure 6B). The cells also appeared freeze‐dehydrated. In untreated mountain pine needles, the transfusion tracheids are clearly perceptible by their bordered pits and their cell walls having an undulate appearance. In frozen samples, the transfusion tracheids are enlarged and appear swollen with stretched cell walls due to ice formation inside. This occurs most likely at the expense of freeze‐dehydration of other cells in the transfusion tissue. In contrast, we could not detect any changes in the shape of the arm palisade parenchyma cells when untreated (20°C Figure 7C), non‐frozen (−2°C, Figure 7D) and frozen samples were compared (−6°C, Figure 7E).

### 3.6 Gas exchange of frozen needles

**FIGURE 8:**
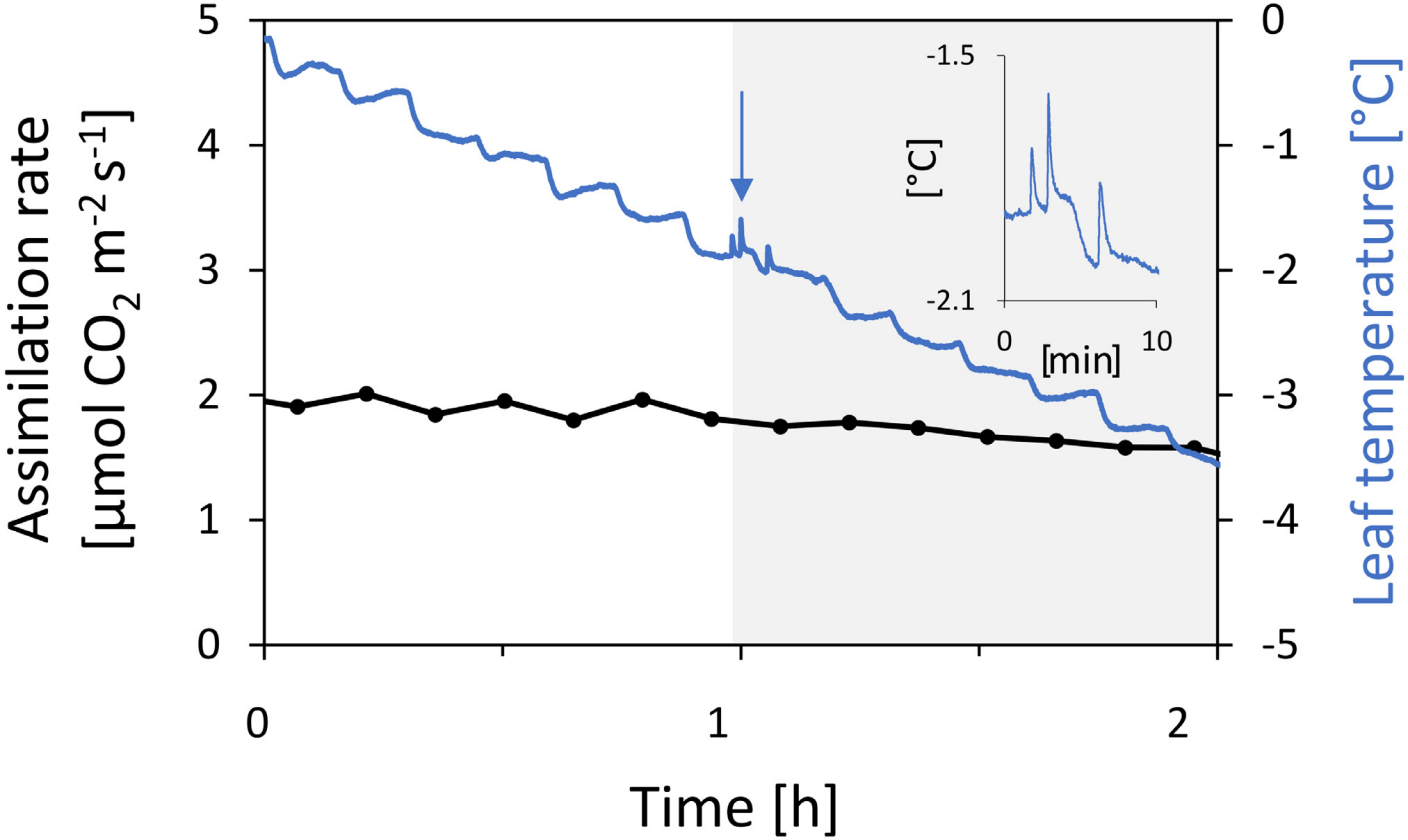
Carbon assimilation rate of mountain pine needles during controlled cooling: Samples were cooled to freezing temperatures at a moderate irradiation of 100 μmol photons m^−2^ s^−1^ photosynthetic photon flux density. Freezing exotherms (arrow) were detected with fine‐wire thermocouples closely attached to the needles and are shown in detail in the inset. Grey shading indicates that the needle was frozen. The carbon assimilation rate was unaffected by ice formation in the needle and continued to decrease with decreasing leaf temperatures. Blue line: Needle temperature, black line: Carbon assimilation rate (filled circles represent mean values of the assimilation rate from six consecutive measurements). The fluctuations seen in the temperature curves result from the stepwise change of the target temperatures during cooling. Needles survived the freezing without any damage as the lowest freezing temperatures reached were much higher than their actual frost‐hardiness.

Gas exchange of mountain pine needles that were successively cooled did not decline abruptly after ice formation (arrow), as exemplarily shown by the carbon assimilation rate in Figure 8. Strikingly, ice formation did not have an immediate effect on gas exchange, which indicates that gas diffusion in the air spaces of the mesophyll is not blocked by ice. In leaves of *Hedera helix* L. and *Helleborus niger* L., ice formed in the intercellular spaces of the spongy tissue (data not shown) and rapidly inhibited gas diffusion. For the frozen mountain pine needles, even 1 h after ice nucleation and a further decrease in freezing temperature, a positive carbon assimilation rate was still recorded.

## 4 DISCUSSION

### 4.1 Lignified endodermis as an apoplastic ice barrier

The physiological function of the endodermis in conifer needles remains unknown (Lersten, 1997; Wu et al., 2005). Our results on mountain pine suggest that the endodermis is a structural ice barrier that controls ice growth by confining ice to the inner central vascular cylinder and the transfusion tracheids. Since no ice was found in the mesophyll, the endodermis blocks ice penetration from transfusion tissue into the mesophyll. The arm palisade parenchyma cells are not freeze‐dehydrated. Consequently, a symplastic water shift towards the endodermis and the iced centre did not occur. As well, no water was shifted to the apoplast. Structural ice barriers, tissues that lack intercellular spaces and have cell walls with narrow pores are impeding the spread of ice (Bertel et al., 2021; Kuprian et al., 2014; Kuprian et al., 2016; Kuprian et al., 2017; Neuner, Kreische, et al., 2019b; Neuner, Monitzer, et al., 2019a). The freezing point declines by a decreasing pore diameter; indeed, when pore diameters are below 6 nm, water can remain unfrozen in cell wall capillaries, which stops the ice wave (Ashworth & Abeles, 1984). The pore diameter is affected by various molecular components but is reduced by suberin and lignin (Chalker‐Scott, 1992), and pectins (Baldwin et al., 2014; Kuprian et al., 2017; Liu et al., 2022; Solecka et al., 2008; Wisniewski & Davis, 1995). These substances have been discussed to impede ice propagation into supercooling buds (Chalker‐Scott, 1992; Kuprian et al., 2017), deep supercooling xylem parenchyma cells (Wisniewski & Davis, 1995) and are important for frost‐hardiness in herbaceous species (Baldwin et al., 2014; Liu et al., 2022; Solecka et al., 2008). The endodermis radial cell walls of mountain pine contained high amounts of lignin. Such radial cell wall modifications are known as Casparian strips in roots. In earlier studies, suberin was suggested as an essential component of Casparian strips, but it is now evident that Casparian strips have a lignin‐based structure (Andersen et al., 2015). The presence of Casparian strips in foliar endodermis is controversially debated (Lersten, 1997; Napp‐Zinn, 1966). A comparison of needle and root Casparian strips in *Pinus bungeana* revealed higher solute permeability for Casparian strips in needles compared to roots, explained by primary pit fields and no suberin in the needle endodermis (Wu et al., 2005). We confirm the absence of suberin in the Raman spectra and could prove the lignin nature. As lignin impregnates radial cell walls and intercellular radial spaces, we suspect that this prominent lignification pattern reduces permeability by smaller pore diameter. This hydrophobisation impedes the apoplastic water flow and might be responsible for blocking the ice spread into the extracellular space of the mesophyll. The control of ice by the endodermis was discussed as a transient interruption of ice propagation by Salt & Kaku (1967) and Kaku (1971), who found a two‐phase freezing exotherm on conifer needles (*Pinus thunbergii*, *Cedrus deodara*). In our experiments, we could not verify such a two‐phase freezing event. However, in our freezing experiments, cooling rates were as in nature; this suggests that the high‐speed freezing protocol employed by Kaku (1971) may have produced such a two‐phase freezing response. Our results are further in contrast to the observations of Roden et al. (2009), who did not find a restriction of ice to the central vascular cylinder in moderately frost‐hardy *Pinus radiata* needles. However, Roden et al. (2009) hypothesised that the endodermis may provide a barrier to water movement towards the ice in the central vascular cylinder.

Mountain pine needles did not show diameter changes during freezing (data not shown); consequently, the shape of needles did not change during freezing, which was similarly observed by Roden et al. (2009). In *P. radiata*, the expansion of water during freezing could be possible as ice accumulated in transfusion tracheids that expanded, whereas transfusion parenchyma cells, Strasburger cells and the endodermis shrank (Roden et al., 2009). A similar freezing response, that is, expansion of transfusion tracheids and shrinkage of the endodermis and transfusion parenchyma cells, was found in mountain pine needles. The endodermis cells contained lipids. Lipids were also detected in spongy cells of *R. glacialis* that are located next to extracellular ice (Stegner et al., 2020b), as also observed in the endodermis. The functional role of lipid accumulation is unknown but hypothesised to be important for controlled water movement to the extracellular ice (Stegner et al., 2020b).

### 4.2 Gas exchange maintained by ice‐free mesophyll

At the alpine treeline, the needles stayed frozen for longer than a month. Consequently, gas exchange appears essential for basic metabolic activity. Early ice nucleation suggests that freezing as such is not avoided. However, the mesophyll remains free of ice. This might allow the ongoing gas exchange since ice in leaves is impermeable to gases (Larcher, 1994). The presence of ice‐free mesophyll is partly in contrast to Roden et al. (2009). In frozen *P. radiata* needles, despite ice in intercellular spaces, distinct gas spaces remained in the mesophyll. Contrarily, in frozen *Eucalyptus pauciflora* leaves, ice filled the intercellular spaces of the spongy tissue, blocking gas exchange immediately (Ball et al., 2002; Ball et al., 2004). Upon ice formation, a similar response was recorded in broadleaved species *H. helix* and *H. niger* with immediate blockage of gas exchange. In overwintering wheat leaves, extracellular ice left very few residual gas spaces (Pearce & Ashworth, 1992). In the summer green nival species *R. glacialis*, ice mass blockage in the spongy tissue is no problem as it lasts only hours during the night (Stegner et al., 2020b). We think mountain pine keeps the intercellular spaces of the mesophyll free of ice to enable gas exchange during prolonged frost periods.

Already before ice nucleation, the photosynthetic rate decreased. This was as well observed for *Pinus sylvestris* and explained by a gradually decreasing mesophyll and stomatal conductance (Lindfors et al., 2015). Stomatal conductance for water vapour decreased with decreasing temperature in the tested species (not shown), but CO_2_ gas exchange was not immediately blocked. In *P. sylvestris*, Lindfors et al. (2015) reported that, CO_2_ assimilation rate and stomatal conductance declined more rapidly upon ice nucleation in needles but were not immediately blocked. Under more severe experimental conditions (ice nucleation −4.3°C, final freezing temperature − 8°C) than in our study (ice nucleation −1.9°C, final freezing temperature − 4°C), after ice nucleation, the assimilation rate was lowered to zero within 17 min and stomatal conductance decreased to a minimum after 45 min. Thereafter, stomatal conductance increased again, leading to the conclusion that the photosynthetic depression was not caused by stomatal closure (Lindfors et al., 2015). Increased internal CO_2_ concentration in frozen needles was attributed to a missing photosynthetic activity (Gaumont‐Guay et al., 2003; Lindfors et al., 2015; Strand & Öquist, 1985) but it could as well be due to uninterrupted respiration. The observation of multi‐mitochondrial networks and autophagic structures in mesophyll cells of frozen needles of mountain pine may be a cell biological indication of altered respiratory physiology (Steiner et al., 2020).

### 4.3 Supercooling of arm palisade parenchyma cells

The invaginations of arm palisade parenchyma cells are air‐filled and thus extensions of the leaf air space system (Wiebe & Al‐Saadi, 1976). In frozen leaves of mountain pine, we found an unaffected shape of the arm palisade parenchyma cells. Consequently, arm palisade parenchyma cells did not freeze‐dehydrate and remained supercooled. Although the mesophyll is ice‐free, symplastic freeze‐dehydration could still be possible towards the endodermis and the frozen central vascular cylinder. Freeze‐dehydration across tissues is known for *R. glacialis* leaves: in the ice‐free palisade tissue, water is shifted via the symplast to the spongy cells and from there released into intercellular spaces (Stegner et al., 2020b). In other herbaceous species, ice forms exclusively in fault zones in the leaf (Kaplenig et al., 2022) or leaf petiole (McCully et al., 2004) and consequently needs to be shifted across tissues or organs.

Why the mesophyll cells of frozen mountain pine needles succeed in maintaining photosynthesis and their shape might be found in microchemistry and structure (Stegner et al., 2022). Arm palisade parenchyma cells are in a strip‐like arrangement between the endodermis and the hypodermis, and tangential cell walls seem to be glued together by lignin. Lignification adds rigidity. Cell wall rigidity is an important feature in cellular water relations and freezing tolerance (Panter et al., 2019; Takahashi et al., 2021). Potentially, rigid cell walls of arm palisade parenchyma cells impede wall deformation. As the endodermis freeze‐dehydrates, there must be either a barrier against shifting water from arm palisade parenchyma cells to the endodermis or a negative tension inside the arm palisade parenchyma cells that is strong enough to keep water against the water potential gradient towards ice. Still, limited space to accommodate ice in the central vascular cylinder may stop any further water movement at a certain point. Overall, supercooling of arm palisade parenchyma cells during freezing seems to be advantageous from a cell physiological perspective as avoidance of freeze‐dehydration may preserve important basic cell physiological functions.

## 5 CONCLUSION

In nature, needles of mountain pine froze around −2°C, could stay permanently frozen for longer than a month and can be subjected to freeze–thaw cycles throughout the whole year. After ice nucleation, ice propagation was uninterrupted from stems into needles due to the lack of ice barriers. Within the needles, the ice masses were strictly confined to the central vascular cylinder. This suggests an important putative function of the endodermis. It is hypothesised that the particular lignification pattern blocks the penetration of ice from the frozen transfusion tissue into the mesophyll. This, together with the structurally rigid mesophyll cell walls, prevented mesophyll freeze‐dehydration. As a result, the intercellular gas spaces of the mesophyll remained free of ice, which may allow needle gas exchange during prolonged periods of frost at the alpine treeline. Similarities in needle anatomy suggest that the results may also apply to other frost‐hardy species of the Pinaceae.

## AUTHOR CONTRIBUTIONS

Gilbert Neuner designed the research. All authors performed experiments. Matthias Stegner, Othmar Buchner, Jasmin Lindner, Alexander Flörl, Nannan Xiao, Andreas Holzinger, Notburga Gierlinger, and Gilbert Neuner analysed the data. Matthias Stegner collected and merged the data. Matthias Stegner, Othmar Buchner, Nannan Xiao, Andreas Holzinger, Notburga Gierlinger, and Gilbert Neuner wrote the manuscript. All authors carefully revised the manuscript.

## CONFLICT OF INTEREST STATEMENT

The authors declare no conflicts of interest.

## Supporting information


**Appendix S1.** Supporting Information.

## Data Availability

The data supporting the findings of this study are available from the corresponding author, Matthias Stegner, upon request.
