# Supplementary material for: Frozen mountain pine needles: The endodermis discriminates between the ice‐containing central tissue and the ice‐free fully functional mesophyll
Source: Physiol Plant. 2023 Feb 8;175(1):e13865. doi: 10.1111/ppl.13865 (PMC10107293; doi:10.1111/ppl.13865)
Supplement: Supplementary file 1 — Appendix S1. Supporting Information. [file PPL-175-0-s001.docx]

## Supporting Information

Article title: Frozen mountain pine needles: The endodermis discriminates between the ice-containing central tissue and the ice-free fully functional mesophyll

Authors: Matthias Stegner, Othmar Buchner, Michael Geßlbauer, Jasmin Lindner, Alexander Flörl, Nannan Xiao, Andreas Holzinger, Notburga Gierlinger, Gilbert Neuner

**Fig. S1** Phloroglucinol stained cross section of a mountain pine needle.

(A) The outer epidermal layer, hypodermis, the radial walls of endodermis and the xylem in the vascular bundles were lignified. (B-C) details viewed by bright-field light microscopy. Abbreviations: APPC arm palisade parenchyma cells, ED endodermis, EP epidermis, HY hypodermis, PH phloem, RC resin channel, ST Strasburger cells, TP transfusion parenchyma, TT transfusion tracheids, XY xylem.


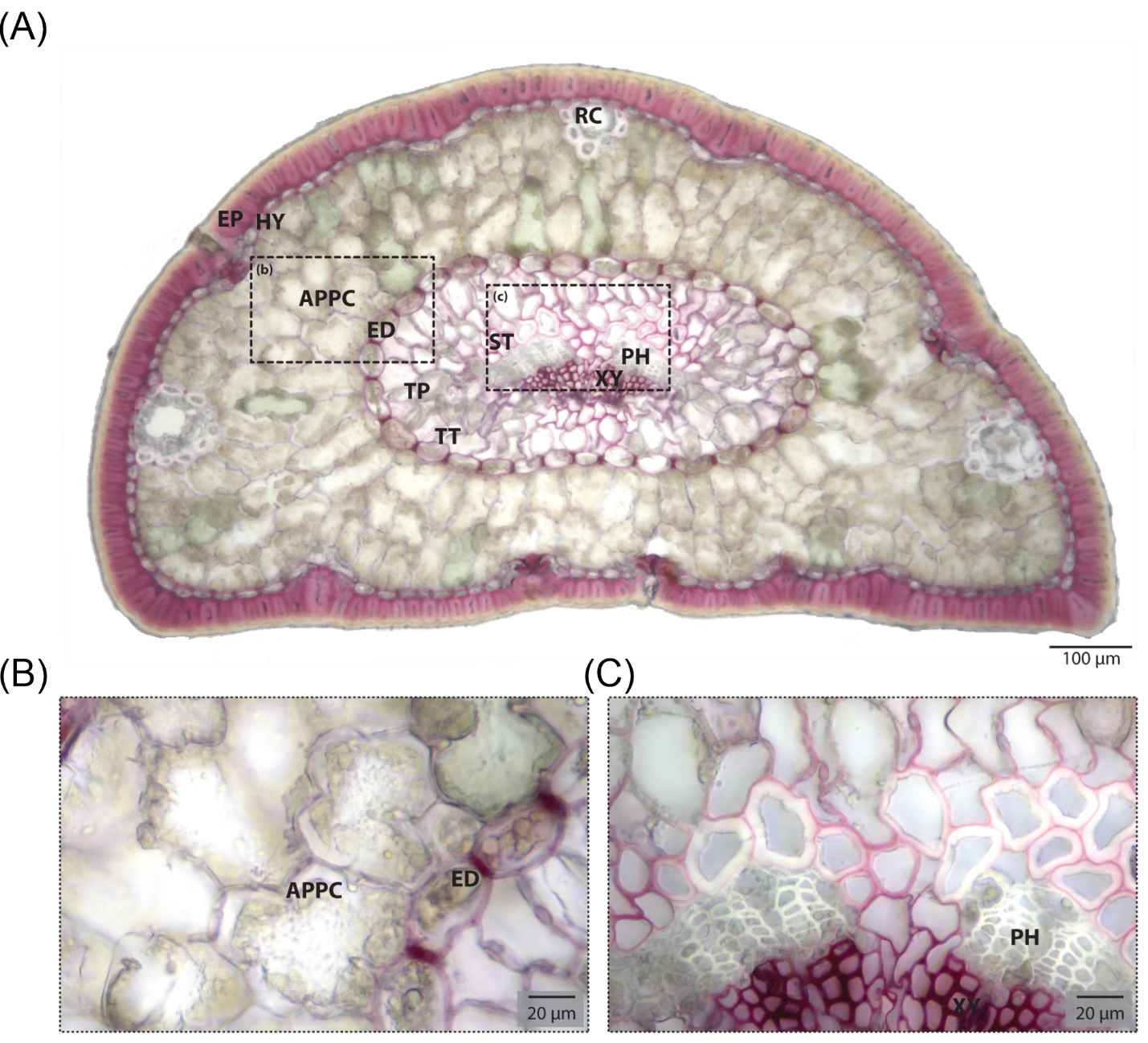


**Fig. S2** Raman imaging based on True Component Analysis.

(A) Overlay of the images: Calcium oxalate monohydrate in green, lipids in yellow, cuticle in orange and chlorophyll in white. (B) Extracting average spectra from the different coloured regions of above image.


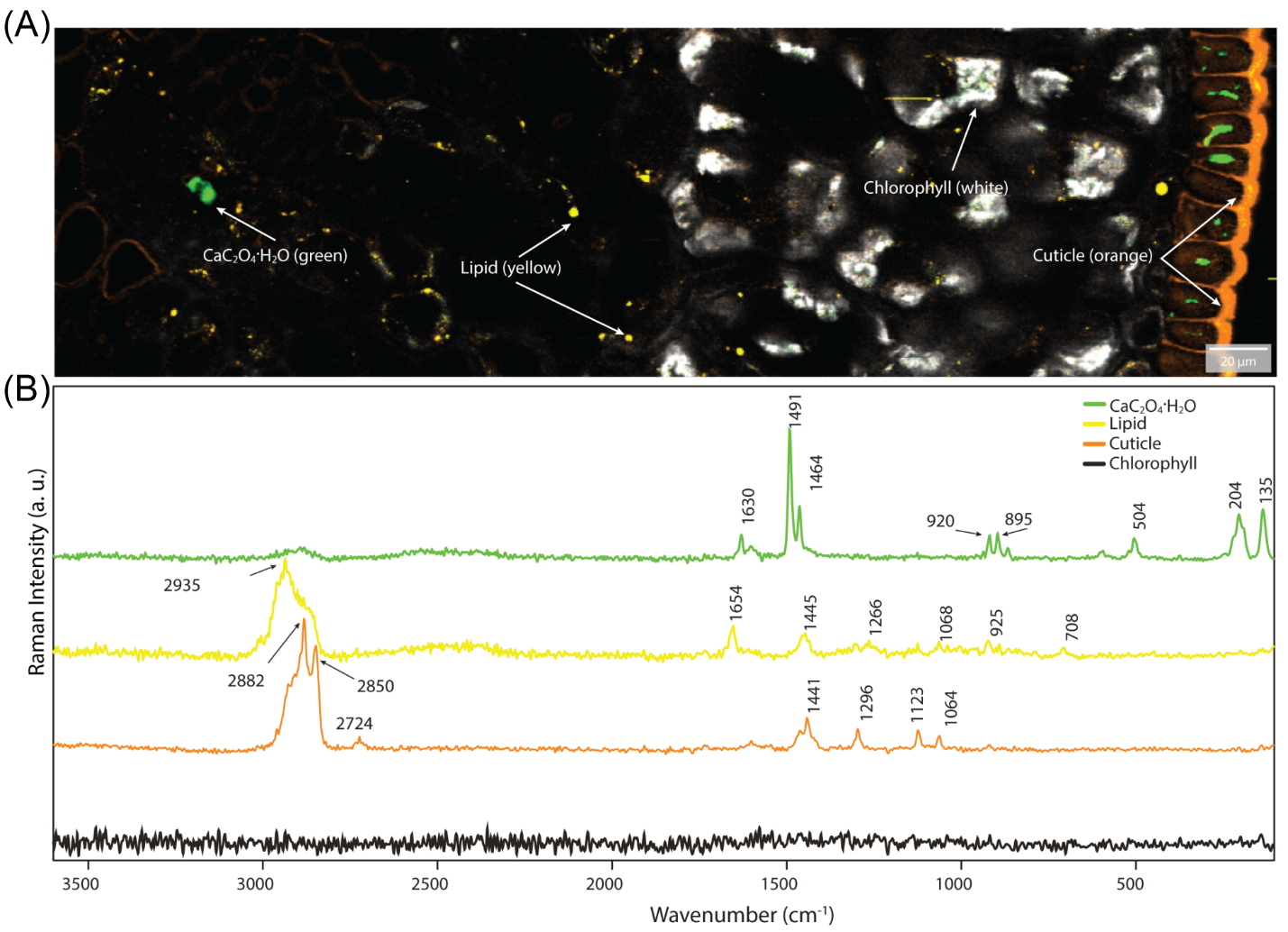


**Fig. S3** Mountain pine needle freezing visualised by infrared thermography.

Spatiotemporal freezing behaviour of mountain pine needles is visualised (A – H) during a controlled freezing treatment. The timestamp indicates the time period since initial ice nucleation in the stem. Additionally, the ambient temperature in the freezing chamber is shown. After nucleation, the ice wave propagated throughout the stem tissue and from there into the attached needles. The overall freezing process lasted approximately 12 minutes (data not shown).

**
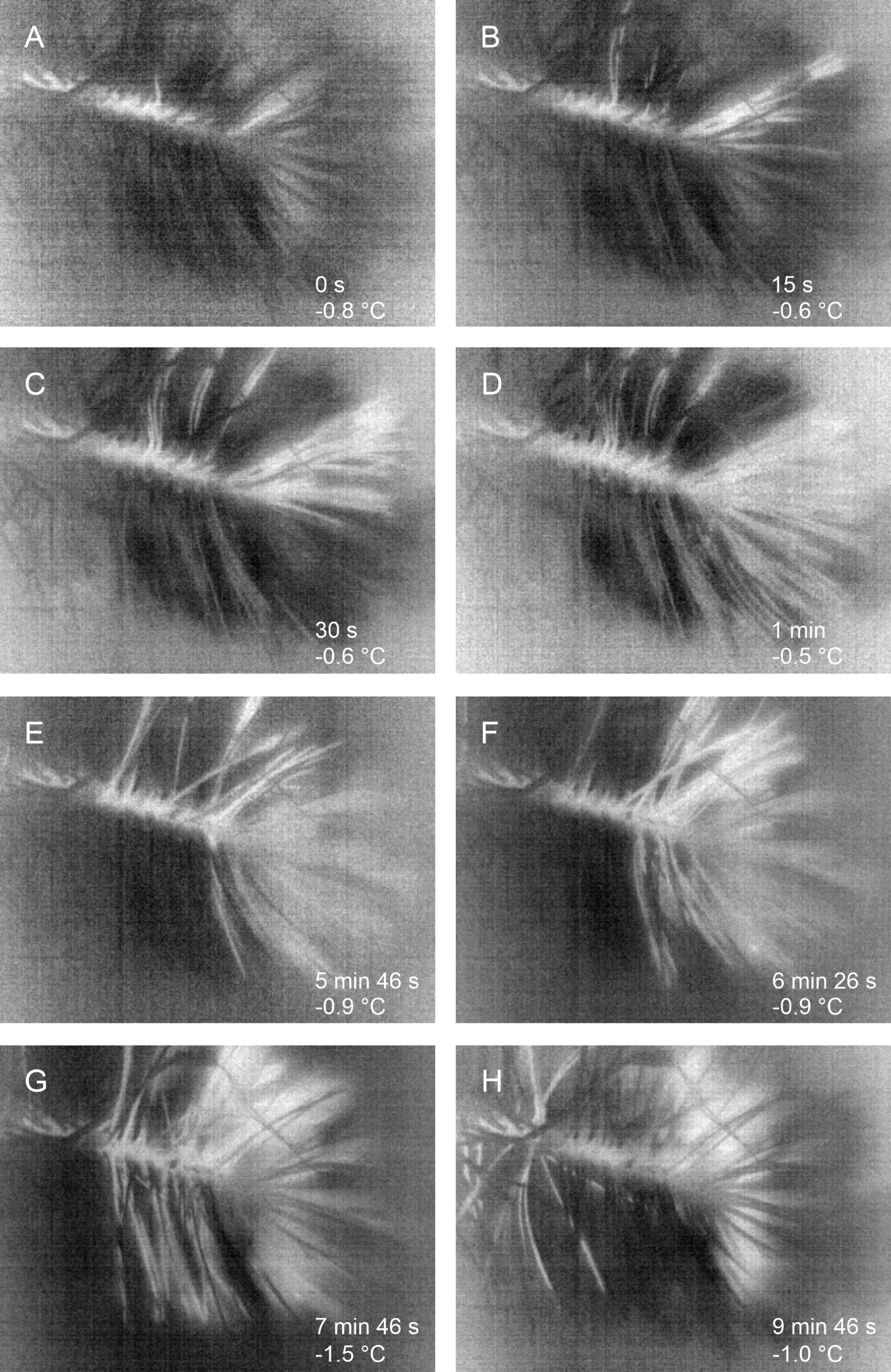
**
